# High‐yield secretion of recombinant proteins from the microalga *Chlamydomonas reinhardtii*

**DOI:** 10.1111/pbi.12710

**Published:** 2017-04-11

**Authors:** Erick Miguel Ramos‐Martinez, Lorenzo Fimognari, Yumiko Sakuragi

**Affiliations:** ^1^ Department of Plant and Environmental Sciences Copenhagen Plant Science Centre University of Copenhagen Frederiksberg C, Copenhagen Denmark

**Keywords:** *C. reinhardtii*, glycomodule, protein secretion, signal sequence, yellow fluorescent protein

## Abstract

Microalga‐based biomanufacturing of recombinant proteins is attracting growing attention due to its advantages in safety, metabolic diversity, scalability and sustainability. Secretion of recombinant proteins can accelerate the use of microalgal platforms by allowing post‐translational modifications and easy recovery of products from the culture media. However, currently, the yields of secreted recombinant proteins are low, which hampers the commercial application of this strategy. This study aimed at expanding the genetic tools for enhancing secretion of recombinant proteins in *Chlamydomonas reinhardtii*, a widely used green microalga as a model organism and a potential industrial biotechnology platform. We demonstrated that the putative signal sequence from *C. reinhardtii* gametolysin can assist the secretion of the yellow fluorescent protein Venus into the culture media. To increase the secretion yields, Venus was C‐terminally fused with synthetic glycomodules comprised of tandem serine (Ser) and proline (Pro) repeats of 10 and 20 units [hereafter (SP)_*n*_, wherein *n* = 10 or 20]. The yields of the (SP)_*n*_‐fused Venus were higher than Venus without the glycomodule by up to 12‐fold, with the maximum yield of 15 mg/L. Moreover, the presence of the glycomodules conferred an enhanced proteolytic protein stability. The Venus‐(SP)_*n*_ proteins were shown to be glycosylated, and a treatment of the cells with brefeldin A led to a suggestion that glycosylation of the (SP)_*n*_ glycomodules starts in the endoplasmic reticulum (ER). Taken together, the results demonstrate the utility of the gametolysin signal sequence and (SP)_*n*_ glycomodule to promote a more efficient biomanufacturing of microalgae‐based recombinant proteins.

## Introduction

The unicellular green microalga *Chlamydomonas reinhardtii* has a long history as a model organism and has helped in the understanding of fundamental biological processes such as metabolism, photosynthesis, phototaxis, chloroplast biology, circadian rhythmicity, cell cycle and mating (Harris *et al*., 2009). As one of the best characterized algal species, *C. reinhardtii* has also been developed as a potential expression platform for the production of recombinant proteins with applications in different industries including biomaterials, bioenergy, therapeutics and nutraceuticals (Almaraz‐Delgado *et al*., 2014; Rasala and Mayfield, 2015; Specht *et al*., 2010). Compared with prokaryotic hosts, recombinant proteins can be expressed from either chloroplast (Purton *et al*., 2013) or nucleus (Jinkerson and Jonikas, 2015) of *C. reinhardtii*, making this microalga a versatile host. Proteins encoded by the nuclear genome can undergo post‐translational modifications (PTMs) and can be targeted to different organelles or the culture media, whereas chloroplast‐expressed proteins are retained inside the plastid. Significant efforts are being made to expand a molecular toolbox, allowing an efficient and robust expression of transgenes from the nuclear genomes of microalgae (Jinkerson and Jonikas, 2015; Mussgnug, 2015). This includes the generation of mutants with increased transgene expression (Neupert *et al*., 2009), codon‐optimized synthetic genes (Fuhrmann *et al.,* 2004; Shao and Bock, 2008), chimeric promoters and use of native introns (Eichler‐Stahlberg *et al*., 2009; Schroda *et al*., 2000), enhanced transgene expression linked to a selection marker (Rasala *et al*., 2012), insertion of promoterless genes fused to an antibiotic‐resistant gene (Díaz‐Santos *et al*., 2016) and recently the use of synthetic promoters for increasing nuclear gene expression (Scranton *et al*., 2016). Moreover, a nuclear episomal vector that is capable of stable replication was recently developed for uses in diatoms (Karas *et al*., 2015).

The secretion of expressed proteins into the medium is an attractive strategy and is widely employed in recombinant protein productions in heterotrophic microbial hosts (Demain and Vaishnav, 2009). In eukaryotes, secretion can ensure proper glycosylation of proteins, which plays important roles in determining the yield, biological activity, stability and half‐life of a secreted recombinant protein (Lingg *et al*., 2012; Mathieu‐Rivet *et al*., 2014). It can also simplify downstream processing and circumvent cost‐ineffective and labour‐intensive cell lysis (Hellwig *et al*., 2004; Nikolov and Woodard, 2004). Moreover, the harvested algal biomass can be exploited as a co‐product, adding more value to the process (Gangl *et al*., 2015). Large‐scale cultivation of transgenic *C. reinhardtii* in photobioreactors has been demonstrated for both wild‐type and cell‐wall‐deficient strains, paving a path towards industrial exploitation of this microalga (Gimpel *et al*., 2015; Zedler *et al*., 2016).

Despite this progress, only a handful of investigations into the secretion of recombinant proteins have been made in microalgae. Thus far, over 30 recombinant proteins have been expressed in *C. reinhardtii*, of which only six have been secreted (Chavez *et al*., 2016; Eichler‐Stahlberg *et al*., 2009; Lauersen *et al*., 2013a,b, 2015b; Rasala *et al*., 2012). Protein secretion is characterized by the presence of a signal sequence that targets a protein to the secretory pathway and ultimately into the culture media. Thus far, four signal sequences have been exploited in fusion with recombinant proteins in *C. reinhardtii*: a non‐native signal sequence from *Gaussia princeps* luciferase (Ruecker *et al*., 2008), native signal sequences from arylsulphatases (ARS1 and ARS2; Eichler‐Stahlberg *et al*., 2009; Rasala *et al*., 2012) and carbonic anhydrase (CAH1; Lauersen *et al*., 2013a). In these cases, the yields of recombinant proteins ranged from 100 μg/L to 10 mg/L (Eichler‐Stahlberg *et al*., 2009; Lauersen *et al*., 2013a; Rasala *et al*., 2012). Interestingly, other species like the diatom *P. tricornutum* are able to secrete up to 2.5 mg/L of a functional human IgG antibody without a signal peptide (Hempel and Maier, 2012), demonstrating how little we know about the secretion mechanism of photosynthetic microorganism. In general, secretion yields above 10 mg/L are considered the minimum for commercial process development (Hellwig *et al*., 2004), and higher yields (>1 g/L) are typically obtained using heterotrophic host organisms. Although extensive optimization of growth parameters has been shown to increase secretion efficiency and productivity in *C. reinhardtii*, reaching up to 12 mg/L (Lauersen *et al*., 2015a), further development is needed to compete with commonly used hosts.

In plant cell cultures, a successful strategy to enhance secretion yields and the stability of recombinant proteins has been demonstrated based on (SP)_*n*_ glycomodules, which are *O*‐glycosylated with arabinogalactan polysaccharides attached to hydroxylated Pro residues (Hyp) (Shpak *et al*., 1999; Xu *et al*., 2007). Briefly, in eukaryotes, many secretory and membrane proteins are glycosylated (Higgins, 2010). This occurs in the secretory pathway starting in ER and becomes elaborated in the Golgi apparatus. Glycosylation serves a variety of structural and functional roles and can be classified into two main categories: *N*‐glycans are linked to the amide group of asparagine residues and *O*‐glycans are linked to the hydroxyl group of Ser, threonine, hydroxy lysine or Hyp residues. It is well established that glycosylation increases the stability of proteins. The presence of *O*‐linked glycans, for instance, strongly enhances the secretion yields and physicochemical properties of a protein by influencing protein folding, solubility, stability and resistance to heat or proteolysis (Gomord *et al*., 2010; Walsh and Jefferis, 2006). In contrast to higher plants, little is known about the mechanisms of protein glycosylation and effects on protein secretion in microalgae (Mathieu‐Rivet *et al*., 2014).

To increase the yield of recombinant proteins in the culture media, we tested whether the putative signal sequence from the metalloprotease gametolysin can efficiently secrete recombinant proteins into the culture media. Using a yellow fluorescent protein, Venus, as a reporter, we confirmed that the gametolysin signal sequence can indeed secrete recombinant Venus. To enhance the secretion yields, we implemented the (SP)_*n*_ glycomodules in *C. reinhardtii*. Venus was expressed as fusion glycoproteins, resulting in up to a 12‐fold increase in the yield in culture media and a greater resistance to proteolytic degradation as compared to the untagged Venus. These results support the potential utilization of transgenic microalgae as a platform for secretion of recombinant proteins.

## Results

### Gametolysin signal sequence targets Venus into the medium

**Figure 1:**
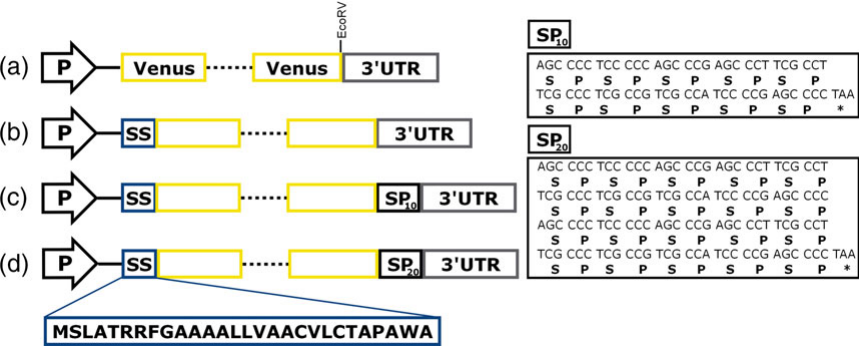
Schematic diagram of the expression cassettes. (a) pERC‐Venus; (b) pERC‐SSVenus; (c) pERC‐SSVenus‐(SP)_10_; (d) pERC‐SSVenus‐(SP)_20_. P, chimeric promoter RBCS2/HSP70A; solid line, RBCS2 intron 1; dotted line, RBSC2 intron 2; SS, gametolysin signal sequence. 3′UTR, BRSC2 terminator. The boxes on the right show the codon‐optimized nucleotide sequences used in this study to encode (SP)_*n*_ fusion tags. The peptide sequence of the gametolysin signal sequence is indicated.

Bioinformatic analysis by SignalP 4.0 (Petersen *et al*., 2011) revealed that the N‐terminal sequence of the metalloprotease gametolysin (Kinoshita *et al*., 1992; Matsuda *et al*., 1987) is highly likely to be a signal sequence (D score of 0.88) and to be cleaved between alanine (position 28) and asparagine (position 29). Therefore, the ability of the N‐terminal 28 residues to secrete a recombinant protein was tested by fusing it to the N‐terminus of Venus. The construct was termed pERC‐SSVenus and consisted of two separate expression cassettes, one for the expression of the gene of interest and another for the expression of an antibiotic resistance marker (paromomycin). As a control, a second construct lacking the signal sequence (pERC‐Venus) was generated (Figure 1). To ensure high levels of expression, we included in our vector design the following genetic elements: the chimeric promoter HSP70A‐RBSC2, RBSC2 intron 1, the RBSC2 intron 2 and the RBSC2 3′ untranslated region (UTR) as a transcriptional terminator as previously used by Eichler‐Stahlberg *et al*. (2009). The expression cassette was introduced into the nuclear genome of the cell‐wall‐deficient mutant UVM4, a strain known for enhanced transgene expression levels (Neupert *et al*., 2009). Integration of the expression cassette was confirmed by colony PCR. Positive cells were screened for either intracellular or extracellular accumulations of Venus by dot blotting analysis using a monoclonal anti‐GFP antibody.

**Figure 2:**
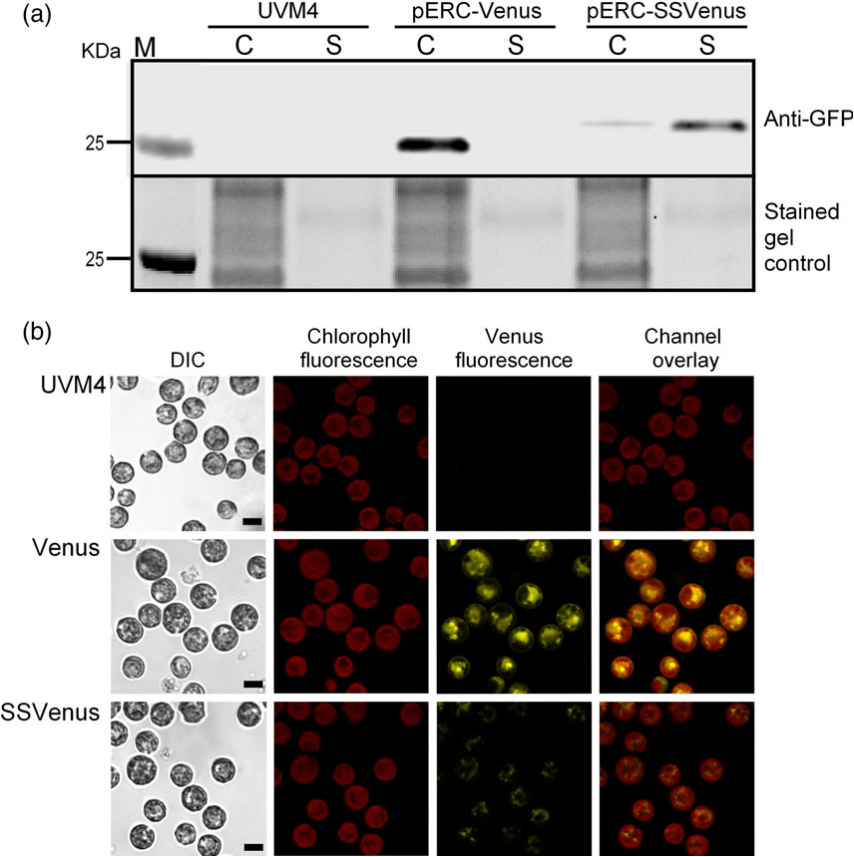
Subcellular localization and expression of fluorescent proteins from the Venus and SSVenus transgenic lines. (a) Western blot analysis of total proteins from cell lysates and supernatants (top panel). Total proteins were loaded in equal amounts and separated by SDS‐PAGE and visualized upon the UV irradiation (lower panel, indicated as ‘stained gel control’). Transgenic lines were grown until ~2 × 10^6^ cells/mL in TAP media. Harvested cells (C) and supernatants (S) were resuspended in 4× Laemmli buffer. The untransformed UVM4 strain was used as a control. (b) Expression of Venus in *C. reinhardtii* cells visualized by confocal microscopy. Parental strain UVM4 was used as a control. Cells transformed with pERC‐Venus (Venus line) and pERC‐SSVenus (SSVenus line) were analysed. All images were acquired using the same settings for all strains. Scale bar indicates 5 μm.

To determine whether Venus was effectively secreted into the culture media, the transgenic lines were grown mixotrophically in tris‐acetate phosphate (TAP) medium. Western blotting of the cell pellets and the cell‐free media were performed using representative lines of the Venus and SSVenus strains generated with the pERC‐Venus and pERC‐SSVenus vectors, respectively. The parental UVM4 strain was used as a control. In the Venus line, Venus was exclusively found in the cell pellets and as expected had an apparent molecular weight of 27 kDa (Figure 2a). In contrast, only a very low amount of Venus was detected in the cell pellets of the SSVenus line, whereas most Venus was found in the media (Figure 2a). Notably, Venus that accumulated in the SSVenus line had a slightly higher molecular weight, by approximately ~2 kDa, both in the cell pellets and in the media. A similar increase in the molecular weight was previously observed for a fungal xylanase when secreted from *C. reinhardtii* (Rasala *et al*., 2012). One possible explanation is that the gametolysin signal sequence is not properly cleaved. Another possible explanation is post‐translational modification such as glycosylation and phosphorylation (Cui *et al*., 2015; Mathieu‐Rivet *et al*., 2013).

Live‐cell imaging was performed by confocal scanning laser microscopy to visualize the localization of the recombinant Venus proteins in the Venus and SSVenus lines (Figure 2b). Non‐transformed cells had no fluorescent signal as expected. The Venus line showed a strong cellular yellow fluorescence signal from active Venus in the cytoplasm confined by the chloroplast, which is consistent with previous reports (Fuhrmann *et al*., 1999; Plucinak *et al*., 2015). In contrast, the SSVenus lines showed weak and punctate fluorescent signals in the cytoplasmic space, corroborating with the Western blot analysis that only a small amount of Venus was present inside the SSVenus cells and is likely to represent Venus in transit through the ER and Golgi before secretion into the culture medium. Taken together, these results demonstrate that the gametolysin signal sequence was able to target Venus to the culture medium.

Subsequently, secretion yields were determined from cell lines with highest relative abundances by dot blotting. To detect Venus, clarified media were concentrated by 10‐fold prior to the analysis and compared with defined amounts of a recombinant Venus that had been expressed and purified from *Escherichia coli*. Supernatants from a Venus line and the parental strain UVM were used as controls. The five independent SSVenus lines, which bear the pERC‐SSVenus construct, were able to secrete the recombinant protein into the media at levels between ~0.3 and 1.3 mg/L (Figure S1). This variation may be attributable to variable transcription activities due to different insertion positions and copy numbers (Jinkerson and Jonikas, 2015).

### Secretion yields of (SP)_*n*_‐fused SSVenus

**Figure 3:**
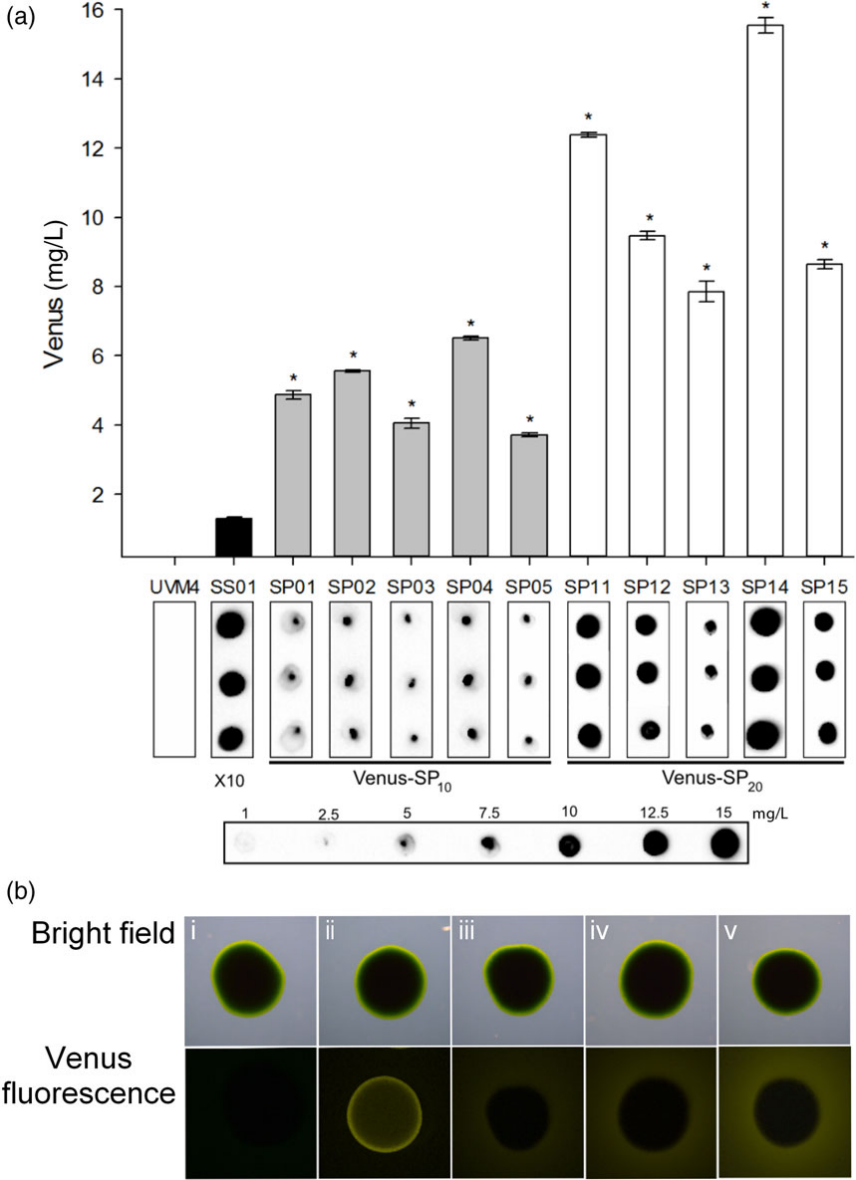
Introduction of (SP)_*n*_ glycomodules enhances the yields of secreted Venus in *C. reinhardtii*. (a) Secretion yields of selected transgenic lines determined by dot blotting. Concentrations of Venus in the culture media from five lines for each construct were quantified using purified *E. coli*‐derived Venus as a standard. Supernatants from SSVenus‐(SP)_10_ and SSVenus‐(SP)_20_ selected lines were collected after 7 days of cultivation and used directly for dot‐blot analysis. Means of three technical replicates and standard errors are shown. Statistical analysis was performed using a one‐way ANOVA (*P* < 0.05). The asterisk represents a significant difference from SS01. (b). Fluorescence emission from cells grown on agar plates detected with a stereo fluorescence microscope. Fluorescence was detected under the same settings for all colonies. i, untransformed UVM4; iii, Venus; iii, SSVenus; iv, SSVenus‐(SP)_10_; v, SSVenus‐(SP)_20_. Scale bars indicate 1 mm.

We aimed to increase the secretion yields by expressing Venus as fusion glycoproteins using synthetic (SP)_*n*_ glycomodules. To this end, SSVenus was fused C‐terminally with the (SP)_10_ and (SP)_20_ glycomodules, giving rise to the vectors pERC‐SSVenus‐(SP)_10_ and pERC‐SSVenus‐(SP)_20_ (Figure 1c, d). These constructs were used to transform the UVM4 strain and transgenic SSVenus‐(SP)_10_ and SSVenus‐(SP)_20_ as described above. Cell‐free culture media were screened by dot blotting using anti‐GFP antibody, and five transgenic cell lines for each construct with the highest relative secretion yield were selected for further quantification. It should be noted that the (SP)_*n*_‐fused SSVenuses were readily detectable in the media of these strains without prior concentration. The highest yields of SSVenus‐(SP)_10_ and SSVenus‐(SP)_20_ fusion proteins in the culture medium were 7.5 mg/L in the line SP04 and 15 mg/L in the line SP14, respectively (Figure 3a). These values are sixfold and 12‐fold higher, respectively, than the secreted SSVenus without the glycomodules. For further analysis, these two lines were selected. When grown on TAP agar plates, a halo of yellow fluorescence surrounding a colony of the SSVenus line was visible, while progressive increases in the fluorescence intensity were seen surrounding the colonies of the SSVenus‐(SP)_10_ and SSVenus‐(SP)_20_ lines (Figure 3b). To our best knowledge, this is the first time that the secretion of recombinant proteins has been enhanced in microalgae by using the (SP)_*n*_ glycomodules.

### (SP)_*n*_‐fused SSVenus are glycosylated

**Figure 4:**
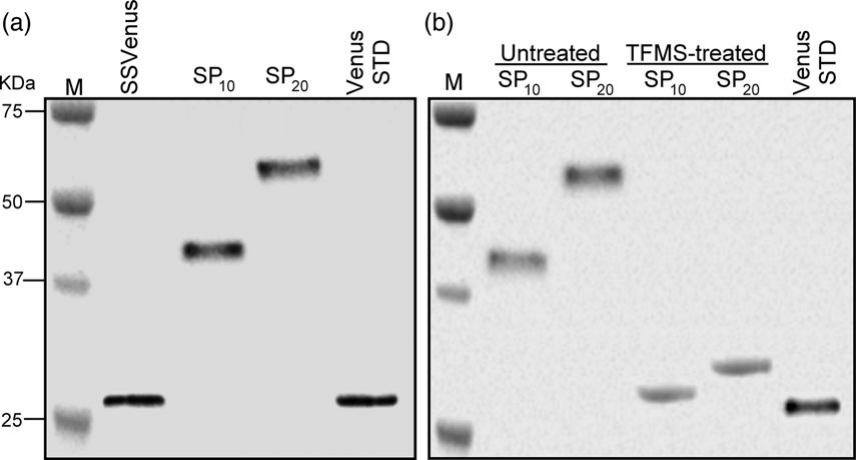
The secreted Venus‐(SP)_*n*_ proteins are glycosylated. (a) Western blot analysis of fusion glycoproteins secreted into the media by the transgenic *C. reinhardtii*. M, molecular size markers. One hundred micrograms of total proteins in the media were loaded in each lane for SSVenus, SSVenus‐(SP)_10_ and SSVenus‐(SP)_20_, whereas 1 μg of purified *E. coli*‐derived Venus was loaded as a control. (b) Effect of deglycosylation treatment with TFMS on the fusion glycoproteins. SSVenus‐(SP)_10_ and ‐(SP)_20_ proteins were chemically deglycosylated and analysed by Western blot. Untreated and TFMS‐treated samples were loaded on parallel lanes for comparison. *E. coli*‐derived Venus, designated as Venus STD, was loaded as a control. M, molecular size markers. SP_10_ and SP_20_ indicate SSVenus‐(SP)_10_ and SSVenus‐(SP)_20_, respectively.

Molecular weights of the SSVenus, SSVenus‐(SP)_10_ and SSVenus‐(SP)_20_ proteins secreted from *C. reinhardii* into the media were analysed by Western blotting. Fusion proteins were detected as single bands at approximately 42 and 53 kDa for SSVenus‐(SP)_10_ and SSVenus‐(SP)_20_, respectively. These values were considerably larger than the sizes of the SSVenus control and the deduced molecular weights of the SSVenus‐(SP)_10_ (28.8 kDa) and SSVenus‐(SP)_20_ (30.7 kDa) (Figure 4a).

We investigated whether SSVenus‐(SP)_*n*_ proteins were glycosylated. The secreted proteins were subjected to chemical deglycosylation by treatment with trifluoromethanesulphonic acid (TFMS), a chemical reagent that efficiently cleaves *N*‐ and *O*‐linked sugars from glycoproteins without affecting the integrity of the polypeptide (Edge *et al*., 1981). The results presented in Figure 4b showed that the TFMS treatment resulted in apparent mass shifts by ~14 and ~23 kDa for SSVenus‐(SP)_10_ and SSVenus‐(SP)_20_ to ~28 and ~30 kDa, respectively, which correspond to the unglycosylated forms of the respective proteins. Therefore, the 42‐ and 53‐kDa forms of (SP)_*n*_‐fused SSVenuses represent glycosylated proteins.

### Kinetics of protein secretion

**Figure 5:**
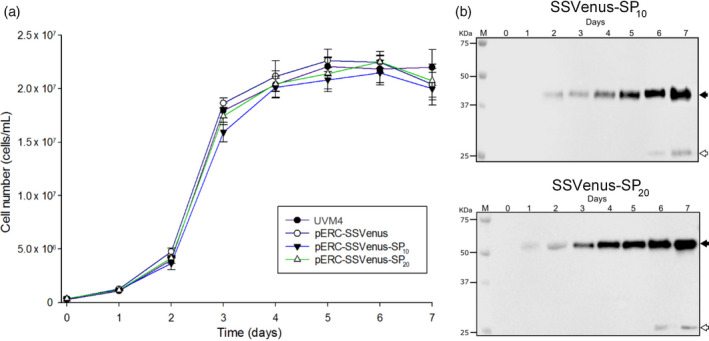
Growth of the transgenic lines of *C. reinhardtii* secreting Venus and fusion Venus is not affected by the production of the recombinant proteins. (a) Growth curves of the transgenic lines and the parental strain UVM4 under the mixotrophic conditions at 25 °C, under constant illumination at 120 μmol photons m^−2^ s^−1^. Strains were inoculated in TAP media at the OD_750nm_ value of 0.05. Growth was monitored by cell counting for 7 days. Error bars represent standard errors of at least three independent cultures. There was no statistically significant difference between genotypes based on two‐way ANOVA test (*P* > 0.05). (b) Time course of Venus‐SP_10_ and Venus–SP_20_ secretions in the growth media. Cell line SP04 expressing SSVenus‐(SP)_10_ (upper panel); cell line SP14 expressing SSVenus‐(SP)_20_ (lower panel). Samples were taken from shake flasks every 24 h for 7 days. Twenty microlitres of clarified supernatants for each sample were separated on SDS‐PAGE and analysed by Western blot. Lanes 1–7 correspond to samples from 0‐ to 7‐day‐old culture supernatants; molecular size markers are indicated as M. Solid arrow indicates the fusion proteins; open arrows represent non‐glycosylated protein.

The accumulation of SSVenus and (SP)_*n*_‐fused SSVenuses in the culture media was monitored for 7 days for the transgenic lines SS01, SP04 and SP14 representing SSVenus, SSVenus‐(SP)_10_ and SSVenus‐(SP)_20_, respectively. The untransformed cells were used as a control. All three transgenic cells grew similarly to the control cells (Table 1; Figure 5a). The average specific growth rates were calculated between 1.29 and 1.48 day^−1^ under the conditions tested (Table 1). Hence, secretion of the recombinant glycoproteins from transgenic lines does not impose an altered metabolic load that can decrease cell growth under the conditions tested. Under these conditions, the maximum yields of the secreted recombinant proteins in the culture media were 1.3, 7.5 and 15.1 mg/L for SSVenus, SSVenus‐(SP)_10_ and SSVenus‐(SP)_20_, respectively, which is consistent with the results described above (Figure 4a). The accumulation of Venus followed the cell growth, and the highest productivity of 2.13 mg/L day was observed for the transgenic cells expressing SSVenus‐(SP)_20_ (Table 1). Interestingly, non‐glycosylated products were present at the end of the cultivation, probably as a result of the spontaneous lysis that occurs when the culture enters the stationary phase and the cells are under stress (Figure 5b).

**Table 1:** Growth parameters and yields of the secreted proteins from transgenic cell lines secreting Venus variants and the parent UVM4 strain

	Maximum biomass (cells/mL)	Specific growth rate (day^−1^)	Doubling time (days)	Maximum yields (mg/L)	Productivity (mg/L/day)
UVM4	2.20 × 10^7^	1.38	0.50	–	–
SSVenus	2.26 × 10^7^	1.29	0.54	1.3	0.19
Venus‐(SP)_10_	2.08 × 10^7^	1.38	0.50	7.7	1.10
Venus‐(SP)_20_	2.25 × 10^7^	1.43	0.48	15.1	2.13

### Effect of Brefeldin A (BFA) on glycosylation of Venuses‐(SP)_*n*_ in the secretory pathway

Despite its importance, subcellular localization of the glycosylated recombinant proteins produced by *C. reinhardtii* has not been well understood at the molecular level. BFA is a fungal toxin that is widely used in studies of eukaryotic secretory pathways, and in *C. reinhardtii*, BFA inhibits the secretory pathway by destroying the Golgi apparatus (Hummel *et al*., 2007). We took advantage of this effect of BFA and tested its impact on the glycosylation of the (SP)_*n*_‐fused Venuses. To do so, we first screened for the effective concentration of BFA on blocking the membrane trafficking. The SSVenus line was grown in TAP media to the mid‐exponential phase (approximately 4 × 10^6^ cells/mL), and the cells were treated with BFA for 4 h at final concentrations of 10, 25 and 50 μm or with dimethyl sulphoxide (DMSO) as a control. Untreated UVM4 strain and the Venus line were also included in the analysis as additional controls. Flow cytometry was used to monitor the changes in the fluorescence signal on the population basis. Distinctive distributions of fluorescence signals as a function of cell sizes were observed for the UVM4 strain, the DMSO‐treated SSVenus line and the Venus line (Figure S2a–c). The BFA treatment of the SSVenus line progressively shifted the population distribution similar to the Venus line (Figure S2). Similar results have previously been reported for the secretion of the arylsulphatase from *C. reinhardtii* (Kagiwada *et al*., 2004). At a concentration of 50 μm, BFA caused more than 95% of cells to accumulate Venus intracellularly (Figure S2d). Importantly, the effect of the BFA treatments was fully reversible. Based on these results, the following experiments were conducted with 50 μm BFA.

**Figure 6:**
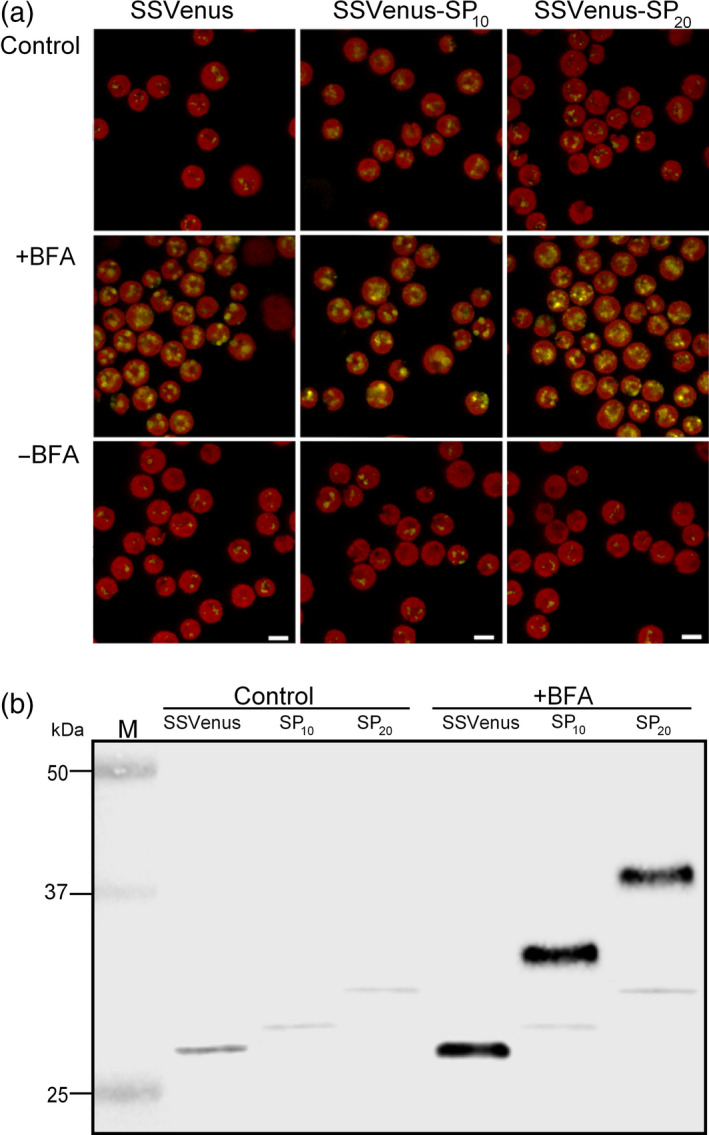
Effect of the BFA treatment on the subcellular localization and molecular masses of the Venus and fusion Venus. Transgenic cells in the exponential growth phase were treated with BFA at 50 μm or solvent control (DMSO) for 4 h. (a) Alteration of the fluorescence patterns in cells secreting Venus and fusion Venus upon treatment with BFA visualized by confocal microscopy. Images show that BFA treatment increases the fluorescence intensity in the transgenic cells as a result of the accumulation in the ER. All images were acquired using the same settings for all strains and treatments. Scale bars indicate 5 μm. (b) Western blot analysis of cell lysates from cells treated with BFA. Lanes 1 and 4, SSVenus; lanes 2 and 5, SSVenus‐(SP)_10_; lanes 3 and 6, SSVenus‐(SP)_20_. One hundred and fifty micrograms of TSP were loaded into each well.

The SSVenus, SSVenus‐(SP)_10_ and SSVenus‐(SP)_20_ lines were treated with 50 μm BFA or DMSO for 4 h, and live‐cell imaging was performed to analyse the localization of SSVenus and SSVenus‐(SP)_*n*_. DMSO‐treated cells of the three transgenic lines displayed weak intracellular signals, consistent with Figures 2 and S2, whereas the cells treated with BFA considerably enhanced the intensities of the intracellular signals in all three lines in a reversible manner (Figure 6a). These results indicate that the BFA treatment successfully blocked the secretion of SSVenus and SSVenus‐(SP)_*n*_, likely by inhibiting the transport from ER to the Golgi apparatus as previously shown in tobacco plants (Boevink *et al*., 1999).

Next, the molecular weights of SSVenus and SSVenus‐(SP)_*n*_ that accumulated intracellularly in BFA‐treated cells were analysed by Western blotting. Firstly, in the absence of the BFA treatment, the SSVenus and SSVenus‐(SP)_*n*_ proteins migrated as single bands with apparent molecular weights that were consistent with the predicted polypeptide backbones, representing non‐glycosylated forms (Figure 6b, Lane 2 and 3). The apparent absence of glycosylated Venus inside the cells suggests that glycosylated forms are more efficiently secreted than the corresponding non‐glycosylated forms. Upon the BFA treatment, additional bands at higher molecular weights were detectable in the SSVenus‐(SP)_10_ and SSVenus‐(SP)_20_ lines (Figure 6b, lane 5 and 6). Their estimated molecular weights were ~32 and ~38 kDa, respectively, and are likely to represent partially glycosylated forms. These results may indicate that *O*‐glycosylation in *C. reinhardtii* starts in the ER (Zhang *et al*., 1989).

### Determination of protein stability

**Figure 7:**
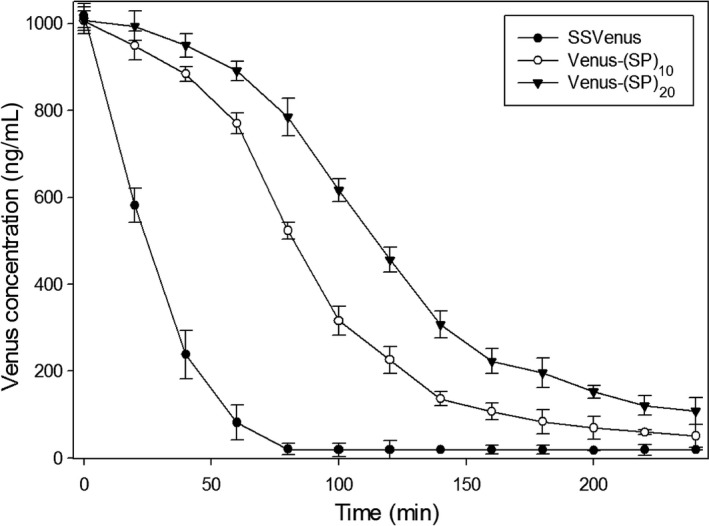
Profiles of proteolytic degradation of secreted fusion proteins. SSVenus, SSVenus‐(SP)_10_ and SSVenus‐(SP)_20_ fluorescence was monitored in the presence of trypsin for 4 h as described in Materials and methods. Quantification of the functional proteins was performed by using the standard curve prepared from purified *E. coli*‐derived Venus. Error bars represent standard errors of at least three independent assays. SSVenus, SSVenus‐(SP)_10_ and SSVenus‐(SP)_20_ were statistically significantly different based on two‐way ANOVA test (*P* < 0.05).

Glycosylation is known to impact the stability of secreted proteins (Solá and Griebenow, 2009). Hence, proteolytic susceptibility of SSVenus‐(SP)_*n*_ was analysed *in vitro*, alongside SSVenus, by monitoring the kinetics of proteolysis with trypsin. SSVenus, SSVenus‐(SP)_10_ and SSVenus‐(SP)_20_ obtained from the respective transgenic culture media were incubated with trypsin for 4 h, and fluorescence was monitored for 240 min. SSVenus was completely degraded by 80 min, with the proteolytic half‐life of 17 min. In contrast, SSVenus‐(SP)_10_ and SSVenus‐(SP)_20_ were found to be significantly more resistant to proteolysis than non‐glycosylated Venus and their complete degradation required >240 min (Figure 7). The proteolytic half‐lives of these proteins were 76 min and 130 min, respectively, which were fourfold and 17‐fold longer as compared to SSVenus. These results demonstrate that Venuses tagged with the (SP)_*n*_ glycomodules are more resistant to proteolytic degradation.

## Discussion

The secretion of recombinant proteins is often desirable not only because it can simplify downstream processing (e.g. easier product recovery), but also because it enables post‐translational modifications, particularly glycosylation, for enhanced product yields and quality. Moreover, the secretion of recombinant proteins could allow direct application of the culture media without extensive purification, which could open up new opportunities in application of microalgae‐derived products. In this study, we firstly showed that the gametolysin signal sequence successfully targeted Venus to the culture media giving rise to the maximum yield of 1.3 mg/L. Secondly, the introduction of synthetic (SP)_*n*_ glycomodules at the C‐terminus of Venus led to an increase in the molecular mass, indicating that the fusion proteins were glycosylated as demonstrated by chemical deglycosylation treatment. Thirdly, the glycomodules considerably increased the proteolytic stability of the fusion proteins. Finally, the yield of the fusion proteins secreted in culture media was enhanced by a 12‐fold to 15 mg/L as compared to the untagged Venus, which is the highest secretion level achieved thus far for microalgae‐based recombinant proteins.

Taken together, these results demonstrate that the gametolysin signal sequence and the synthetic glycomodules can be used to secrete recombinant proteins with higher yields than previously attained and can be added to the growing molecular toolbox in the engineering of *C. reinhardtii* and possibly other microalgae. To establish microalgae as alternative hosts for protein secretion, further enhancement in the yield of secreted proteins is needed. Increasing the length of the synthetic (SP)_*n*_ glycomodule (Zhang *et al*., 2016) has been shown to dramatically increase yields of secreted proteins in plants. A rigorous optimization of culturing conditions, for example by varying operating conditions of a photobioreactor, has been shown to increase the yield of a secreted recombinant protein in *C. reinhardtii* (Lauersen *et al*., 2015a). These optimization strategies may be applied in combination with the use of new mutants with improved transgene expression (Kong *et al*., 2015; Kurniasih *et al*., 2016) and genetic tools such as codon optimization (Barahimipour *et al*., 2015) and novel synthetic algal promoters with increased performance (Scranton *et al*., 2016).

The (SP)_*n*_ glycomodules were originally developed for secretion of recombinant proteins from plant cell cultures, leading to extensive *O*‐glycosylation and enhanced proteolytic stability of the expressed proteins (Shpak *et al*., 1999; Xu *et al*., 2007, 2008, 2010). From a biotechnological point of view, glycosylation of recombinant proteins is a desirable feature in recombinant protein secretion because it can strongly influence the physiochemical properties of a protein, such as folding, solubility, stability, biological activity and resistance to heat or proteolysis (Gomord *et al*., 2010; Mathieu‐Rivet *et al*., 2014; Walsh and Jefferis, 2006). Glycosylation of recombinant proteins (i.e. *xylanase 1* from *Trichoderma reesei*, a human erythropoietin, ice‐binding protein) secreted by *C. reinhardtii* has been speculated to occur in previous studies (Eichler‐Stahlberg *et al*., 2009; Lauersen *et al*., 2013b; Rasala *et al*., 2012); however, detailed experimental evidence supporting the notion has been lacking. Our results show that the glycomodule can be readily applied as a modular unit to enhance the yield and proteolytic stability of recombinant proteins in *C. reinhardtii*. This strategy can be applied either alone or in combination with other strategies to further increase the secretion yield (Lauersen *et al*., 2015a), thereby paving a path towards the development of cost‐effective production of recombinant proteins.

The exact nature of glycoconjugates attached to Venus‐(SP)_*n*_ glycoproteins expressed in *C. reinhardtii* is yet to be elucidated. In plants, the Hyp‐*O*‐glycosylation code was established using synthetic glycomodules based on Hyp‐rich glycoproteins (HGRPs), which are major components of the cell wall of plants and some microalgae including *C. reinhadtii* (Showalter, 1993; Woessner and Goodenough, 1994). Briefly, after hydroxylation of Pro residues, *O*‐Hyp glycosylation is defined by a glycomodule. Branched and structurally heterogeneous arabinogalactan polysaccharides (AGPs) are added via a β‐galactosyl glycosidic linkage to a glycomodule consisting of noncontiguous Hyp repeats [as in the (SP)_*n*_ glycomodules], which is a common feature amongst arabinogalactan proteins (Shpak *et al*., 1999; Tan *et al*., 2003, 2004). On the other hand, a glycomodule consisting of contiguous repeats of Pro and Ser (e.g. Ser‐Pro_4_ repeats), widely conserved amongst extensin proteins, is decorated at the Hyp residues with simple arabinooligosaccharides of typically four monosaccharide units via a β‐arabynosyl glycosidic linkage (Kieliszewski and Shpak, 2001; Shpak *et al*., 2001). [Correction added on 28 June 2017, after first online publication: In the preceding sentences, “α‐galactosyl” and “α‐arabinosyl” were previously incorrect and this has been corrected in this current version.] The HRGPs present in members of Chlorocalles and Volvocales including *C. reinhardtii* contain contiguous and noncontiguous Hyp repeats (Voigt *et al*., 2014; Woessner and Goodenough, 1989, 1992). Some of the Hyp‐bound glycoconjugates characterized to date in *C. reinhardtii* are relatively short, ranging in size and composition from single sugars (arabinose or galactose) to arabinooligosaccharides similar to extensins or arabinogalacto‐oligosaccharides consisting of up to six monosaccharide units (Bollig *et al*., 2007; Ferris *et al*., 2001; Miller *et al*., 1972). Hence, it is plausible that the (SP)_*n*_ glycomodule expressed in *C. reinhardtii* is also glycosylated with relatively short oligosaccharides.

Subcellular localization of *O*‐glycan biosynthesis in higher plants is beginning to emerge, while it is still poorly understood in microalgae. Concerning the extensin‐type *O*‐glycosylation in *C. reinhardtii*, Pro residues are first hydroxylated by prolyl‐4‐hydroxylases localized in the ER (Keskiaho *et al*., 2007), and then, arabinosylation of the Hyp residues in the ER and Golgi apparatus by Hyp‐*O*‐arabinosyltransferases (HPATs) occurs (Zhang and Robinson, 1990; Zhang *et al*., 1989). In plants, these processes appear to take place in the Golgi apparatus (Ogawa‐Ohnishi *et al*., 2013), although evidence suggests that it may also occur in the ER (Estevez *et al*., 2006). Concerning the AGP‐type *O*‐glycosylation, it has been shown in plants that Hyp‐galactosyltransferases (HPGT1 through HPGT3 and GALT3 through GALT6) localize to the Golgi apparatus (Basu *et al*., 2013; Ogawa‐Ohnishi *et al*., 2013), while GALT2 was shown to localize to both the ER and the Golgi apparatus (Basu *et al*., 2013). In this study, the BFA treatment that blocks protein transport from ER to the Golgi apparatus led to the accumulation of partially glycosylated forms of (SP)_*n*_‐fused Venus in ER (Figure 6), which supports the notion that *O*‐glycosylation starts in the ER (Zhang *et al*., 1989). In Arabidopsis, three HPATs involved in Hyp arabinosylation have been identified and shown to localize to the *cis*‐Golgi (Ogawa‐Ohnishi *et al*., 2013). Genome‐wide search using the BLAST database for Arabidopsis HPATs homologues in *C. reinhardtii* identified three putative proteins (Cre01.g032600, Cre12.g531450, Cre16.g690000) that contain putative N‐terminal transmembrane domains and share 20%–33% amino acid identities with Arabidopsis HPATs. In contrast, a genome‐wide search for homologous proteins to plant Hyp‐galactosyltransferases (HPGTs, GALT2) failed to identify a significant hit (E value <0.001), suggesting that the mechanism of Hyp‐galactosylation may be different between *C. reinhardtii* and plants.

In summary, microalgae offer great potential as a light‐powered and low‐cost platform for the secretion of recombinant proteins. We demonstrated that a plant‐based system to enhance protein secretion can be readily transferred to *C. reindhardtii*. Therefore, it can potentially be applied to other algae species (Hempel and Maier, 2012). A further investigation into glycosylation processes would be beneficial for a better understanding of the underlining molecular mechanisms of *O*‐glycosylation in microalgae. The set of synthetic glycomodules established in this study could facilitate this investigation. The molecular tools presented here allow a high‐yield secretion of glycosylated proteins with a reduced susceptibility to proteases. These qualities are of biotechnological importance to minimize downstream processing costs.

## Experimental procedures

### Materials

All standard chemicals and reagents were purchased from Sigma‐Aldrich, USA. Restriction enzymes were purchased from New England Biolabs, USA. All the oligonucleotides were purchased from Integrated DNA Technologies, Inc., USA.

### Assembly of transformation vectors

Venus gene [a super enhanced YFP (Nagai *et al*., 2002)] was codon‐optimized *in silico* based on the codon usage of the nuclear genome of *C. reinhardtii* using the IDT Codon Optimization tool (https://eu.idtdna.com/CodonOpt). The codon‐optimized Venus gene was synthesized in fusion with the sequence of the gametolysin signal sequence consisting of 28 amino acids (Kinoshita *et al*., 1992) at the 5′ end, and an EcoRV restriction site was included at the 3′ end followed by a stop codon (Integrated DNA Technologies, Inc.). In the subsequent cloning, DNA parts were amplified by PCR using Phusion High‐Fidelity DNA Polymerase (New England Biolabs) with a set of overlapping primers to generate pERC‐Venus (Hcr123F, Hcr123R, Venus1F, Venus1R, RBSC2i2F, RBSC2i2, Venus2F, Venus2R) and pERC‐SSVenus (Hcr123F, Hcr123R, SSVenus1F, SSVenus1R, RBSC2i2F, RBSC2i2, Venus2F, Venus2R; where SS denotes gametolysin signal sequence; Table S1). The PCR products were purified and prepared for Gibson Assembly using Gibson Assembly Master Mix (New England Biolabs). Vector pER123 was used as plasmid backbone; it contains the HSP70/RBCS2 chimeric constitutive promoter (Schroda *et al*., 2000), the 3′ UTR from RBSC2 as a terminator and the APHVIII resistance gene for selection on paromomycin, whose expression is controlled by the constitutively PSAD promoter. The final constructs are shown in Figure 1.

A synthetic gene encoding ten repeats of the dipeptide Ser‐Pro was constructed from two codon‐optimized and complementary oligonucleotides (SP10‐F and SP10‐R, Table S1) synthesized with 5′ phosphorylation. Three extra bases (ATC) at the 5′ end of the double‐stranded fragment were added to restore the EcoRV site (Table S1). The oligonucleotides were resuspended in an annealing buffer (100 mm potassium acetate; 30 mm [4‐(2‐hydroxyethyl)‐1‐piperazineethanesulphonic acid (HEPES), pH 7.5] and mixed in equal molar amounts. The mixed oligonucleotides were incubated at 94 °C for 2 min in a water bath and allowed to cool down for 1 h at room temperature. The resulting double‐stranded synthetic gene was inserted into EcoRV‐digested and dephosphorylated pERC‐SSVenus to generate pERC‐SSVenus‐(SP)_10_. Vector pERC‐SSVenus‐(SP)_20_ was constructed by ligation of the annealed oligonucleotides with EcoRV‐digested and dephosphorylated pERC‐SPVenus‐(SP)_10_. The extra nucleotides introduced in pERC‐SSVenus‐(SP)_20_ for cloning purposes were removed via site‐directed mutation using the Q5 Site‐Directed Mutagenesis Kit (New England Biolabs) using primers mSP20‐R and mSP20‐F. Transformation of *E. coli* strain TOP10, plasmid isolation and confirmation of the DNA sequence were according to standard protocols.

### Culture conditions

The cell‐wall‐deficient UVM4 strain of *C. reinhardtii* (Neupert *et al*., 2009) was cultivated in flasks containing TAP media (Gorman and Levine, 1965) at 25 °C under constant illumination (120 μmol photons m^−2^ s^−1^) using cool fluorescent white light with constant agitation at 120 rpm in an orbital shaker. Cell concentrations in cultures were determined by counting the cell number in a Neubauer hemocytometer under a bright‐field microscope. For each sample, three biological replicates were analysed. For quantification of Venus and Venus fused with the glycomodules, cells were grown to exponential or late exponential phase under the conditions indicated above and samples were taken every 24 h. When designated, cultures were grown on TAP agar plates, which consist of TAP media containing 1.5% (w/v) agar.

### Transformation of *C. reinhardtii*

*Chlamydomonas reinhardtii* UVM4 was transformed according to the glass bead method (Kindle, 1990), using 1 μg of a designated vector linearized with ScaI. Cells were incubated overnight and were harvested at 1100 × ***g*** for 5 min and plated on TAP agar plates containing 10 μg/mL paromomycin. After 7 days of incubation, paromomycin‐resistant colonies were picked and further cultivated to be screened for gene integration by colony PCR as described by Cao *et al*. (2009) using gene‐specific primers GSP3 and GSP4 to amplify the full‐length expression cassette (Table S1).

### Immunoblotting

To detect the expression of Venus and Venus fused with the glycomodules, cultures of the transformed cell lines were subjected to centrifugation at 5000 × ***g*** for 5 min at 25 °C. Pellets were resuspended in 4× Laemmli buffer (BioRad, Copenhagen, Denmark) and denatured at 90 °C for 5 min. Supernatants were either used directly for analysis or concentrated 10‐fold by freeze drying and denatured in the presence of 4× Laemmli buffer as described above. Either lysates and/or supernatants were subjected to protein quantification and detection by dot blotting or Western blotting. For Western blotting, samples were loaded on a 12% (w/v) Criterion™ TGX Stain‐Free™ Protein Gel, which contains trihalo compounds that can react with tryptophan residues under the UV irradiation and gives rise to fluorescence (BioRad). Separated proteins were transferred to a polyvinylidene difluoride membrane (PVDF). For dot blotting, 1 μL of supernatant was spotted on a nitrocellulose membrane and left to dry at room temperature for 1 h; this was performed in technical triplicates for every positive strain. Purified *E. coli*‐derived Venus was used as a standard for protein quantification or as a positive control. Immunodetection of Venus was carried out using anti‐GFP mouse IgG (Roche Applied Science, Germany) in a 1 : 2000 dilution according to the manufacturer's instructions. No cross‐reactivity with the proteins native to *C. reinhardtii* was detected by the anti‐GFP antibody under the experimental conditions employed in this study. The secondary antibody (anti‐mouse, IgG secondary antibody, horseradish peroxidase conjugate, Sigma‐Aldrich) was used in a 1 : 2500 dilution. Total soluble protein concentration was measured using Micro BCA™ Protein Assay Kit according to the manufacturer's instructions (Thermo Fisher Scientific, Hvidovre, Denmark).

### Fluorescence localization analysis

Live‐cell imaging was performed using a Leica SP5‐X confocal laser‐scanning microscope with a 63× water‐immersion objective. Localization of Venus was imaged using a 514 nm argon laser; fluorescence emission was detected between 520 and 550 nm. Chlorophyll fluorescence was observed independently upon excitation at 488 nm and emission 650–700 nm. All images were acquired using the same settings and were analysed by the Leica Application Suite Advanced Fluorescence software.

For direct fluorescence detection on TAP agar plates, 10 μL of cells was spotted on TAP agar plates containing 10 μg/mL paromomycin and incubated under standard growth conditions, as indicated above, for 4 days. Algal colonies grown on the TAP agar plates were imaged by a fluorescence stereo microscope (Leica MZ FLII, Germany) using UV light source (Leica DN 5000B, Germany) using an excitation filter 470/40 nm and an emission filter of 525/50 nm.

### Chemical deglycosylation

Chemical deglycosylation by TFMS was performed as previously described (Edge *et al*., 1981) with a modification. Briefly, cells were removed from cultures by centrifugation at 5000 × ***g*** for 5 min, and the cell‐free supernatants were desalted and lyophilized. One milligram of desalted and lyophilized cell‐free supernatants containing the secreted Venus and (SP)_*n*_ tagged Venus was mixed with 1 mL of a 9 : 1 (v/v) mixture of TFMS and anisole in a glass vial. The mixture was incubated at −80 °C for 5 min and then at −20 °C for 4 h; thereafter, it was neutralized with 1 mL of ice‐cold 60% (v/v) aqueous pyridine solution followed by the addition of 100 μL of 100 mm NH_4_HCO_3_, pH 8.0. Deglycosylated proteins were recovered by centrifugation at 15 000 × ***g*** for 10 min at 4 °C; after centrifugation, the pellet was dissolved in 4× Laemmli buffer, as indicated above. Samples were analysed by Western blot as described above.

### BFA treatment

BFA was dissolved in DMSO at a concentration of 10 mg/L and stored at −20 °C until use. For BFA treatment, cells cultures were grown to mid‐exponential phase (4 × 10^6^ cells/mL) in 24‐well plated as indicated above. An aliquot of this stock solution was added to cell culture to obtain final concentrations of 10, 25 and 50 μm. The same volume of DMSO was added to make solvent control samples. The cultures with BFA were incubated for 4 h. For recovery experiments, the cells were further incubated for 4 h with BFA‐free TAP media. After each treatment, 200 μL of cell cultures was transferred into a 96‐wll plate and was analysed using a BD LSR Fortessa flow cytometer (DB, USA) fitted with multiwell plate sampling system using a YFP filter (excitation at 488 nm; emission at 545/35 nm). Fluorescence was recorded from ten thousand cells per sample. Data were collected, transformed to the logarithmic scale and analysed using BD FACSDiva™ v6.2 software. Cell imaging was performed on cells treated with 50 μm BFA and cells recovered as previously described. Western blotting was carried out on lysates of cells treated with 50 μm BFA as described above. DMSO‐treated cells were used as control in all experiments.

### Proteolytic assay for protein stability

Concentrations of the secreted Venus and (SP)_*n*_ tagged Venus were adjusted using a standard curve prepared from *E. coli*‐expressed Venus. Proteolysis assay was performed using trypsin at a ratio of 1 : 100 (trypsin : Venus) in a trypsin buffer (67 mm sodium phosphate buffer, pH 7.6). Each recombinant protein was analysed in triplicates in 96‐well black flat‐bottom plates. Proteolytic reactions were incubated at 25 °C, and fluorescence was monitored every 20 min for 4 h using a TriStar² LB 942 Multidetection Microplate Reader (BERTHOLD, Germany; excitation 510 nm, emission 550 nm). TAP media were used to determine the baseline.

### Statistical analysis

In all experiments, three biological or technical replicates were performed for each sample and the mean value represents the average. Statistical analysis was carried out using the ANOVA test.

## Conflict of interest

The authors declare no conflicts of interest.

## Supporting information


**Figure S1** Secretion yields of selected SSVenus transgenic lines as determined by dot blotting.
**Figure S2** Analysis of the impact of the BFA treatment on the SSVenus strain by flow cytometry.
**Table S1** List of oligonucleotides used in the study.
