# Supplementary material for: High‐yield secretion of recombinant proteins from the microalga Chlamydomonas reinhardtii
Source: Plant Biotechnol J. 2017 Apr 11;15(9):1214–24. doi: 10.1111/pbi.12710 (PMC5552477; doi:10.1111/pbi.12710)
Supplement: Supplementary file 1 — Figure S1 Secretion yields of selected SSVenus transgenic lines as determined by dot blotting. Figure S2 Analysis of the impact of the BFA treatment on the SSVenus strain by flow cytometry. Table S1 List of oligonucleotides used in the study. [file PBI-15-1214-s001.docx]

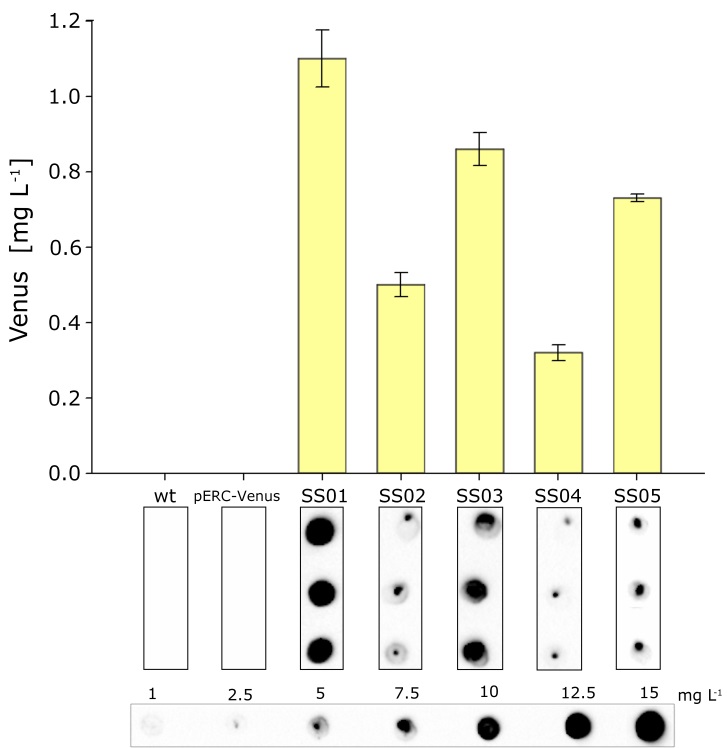


**Figure S1.** Secretion yields of selected SSVenus transgenic lines as determined by dot blotting. Concentrations of SSVenus in the culture media from five independent lines were quantified using purified *E. coli*-derived Venus as a standard. Supernatants from Venus and SSVenus lines were collected after 7 days of cultivation, concentrated ten-fold and used for dot-blot analsysis. Means of three technical replicates and standard errors are shown.


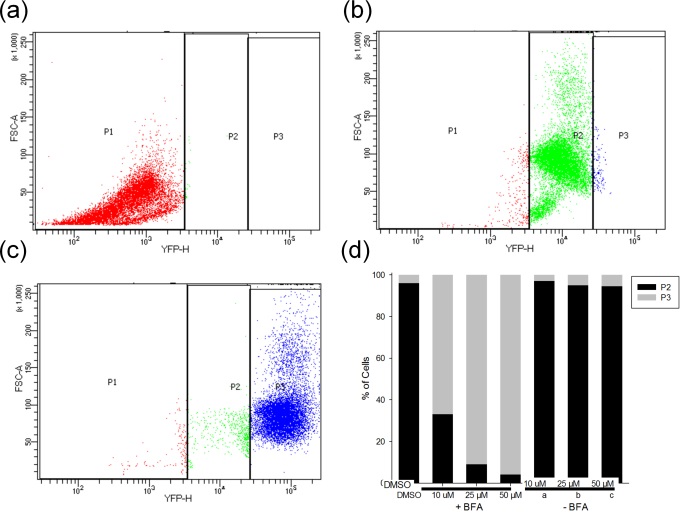


**Figure S2.** Analysis of the impact of the BFA treatment on the SSVenus strain by flow cytometry. For each cell lines and treatment, 10,000 cells were analysed. (a) parental strain UVM4; (b) trangenic SSVenus line, and (c) transgenic Venus line expressing Venus in the cytosol. These strains were used to define regions designated as P1, P2, and P3, which are specific for no yellow fluorescence, cells secreting Venus, and cells accumulating Venus intracellularly, respectively. (d) Bar graph showing the percentages of the populations that were in the P2 and P3 regions after 4 hours of BFA addition (+BFA) and subsequent recovery in the absence of BFA (-BFA). DMSO-treated cells were used as a control.

**Supplementary Table 1.** List of oligonucleotides used in the study.

EcoRV site is shown in bold. Ten Ser-Pro tandem repeats are shown underlined.

| Primer Name | Primer sequence (5’ – 3’) |
| --- | --- |
| Hcr123F | CTGCAAATGGAAACGGCG |
| Hcr123R | CCGCTCCGTGTAAATGGAG |
| Venus1F | GTCGCCGTTTCCATTTGCAGGTGAGCAAGGGCGAGGAG |
| Venus1R | GCAAGCTCACCGTCGTCCTTGAAGAAGATGG |
| SSVenus1F | GTCGCCGTTTCCATTTGCAGATGTCGCTGGCGACGCGG |
| SSVenus1R | GCAAGCTCACCGTCGTCCTTGAAGAAGATGGTGC |
| RBSC2i2F | AAGGACGACGGTGAGCTTGCGGGGTTGC |
| RBSC2i2R | TTGTAGTTACCTGCAAGCAAGGGGATGAAG |
| Venus2F | TTGCTTGCAGGTAACTACAAGACCCGCG |
| Venus2R | CCTCCATTTACACGGAGCGGTTAGATATCCTTGTACAGCTC |
| SP10-F | **ATC**AGCCCCTCCCCCAGCCCGAGCCCTTCGCCTTCGCCCTCGCCGTCGCCATCCCCGAGCCCC |
| SP10-R | GGGGCTCGGGGATGGCGACGGCGAGGGCGAAGGCGAAGGGCTCGGGCTGGGGGAGGGGCT**GAT** |
| mSP20-F | AGCCCCTCCCCCAGCCCG |
| mSP20-R | GGGGCTCGGGGATGGCGAC |
| GSP3 | CTA GAA CTA GTG CTG AGG CTT G |
| GSP4 | CGA AGG ATC CCG CTT CAA ATA C |
